# Dr. Chester W. Stranahan

**Published:** 1911-05

**Authors:** 


Dr. Chester W. Stranahan, of Erie, Pa., died at his home April 6, 1911, from paralysis, aged 65 years. He received his medical degrees from the University of Buffalo in 1863, and from Jefferson Medical College in 1867. He was surgeon in charge of St. Vincent's Hospital, Warren, from 1892 to 1902, and for a number of years was local surgeon of the Lake Shore and Michigan Southern Railway Company.

